# Neoadjuvant Therapies for Patients with Locally Advanced Gastric Cancer: A Retrospective Cohort Study*

**DOI:** 10.5152/eurasianjmed.2024.24468

**Published:** 2024-06-01

**Authors:** Enes Ağırman, Yavuz Albayrak, Rıfat Peksöz, Mesud Fakirullahoğlu, Furkan Ali Uygur, Esra Dişçi, Mehmet İlhan Yıldırgan, Sabri Selçuk Atamanalp

**Affiliations:** 1 Department of General Surgery, Erzurum City Hospital, Erzurum, Türkiye; 2 Department of General Surgery, Ataturk University Faculty of Medicine, Erzurum, Türkiye; 3 Department of General Surgery, Giresun University Faculty of Medicine, Giresun, Türkiye

**Keywords:** Gastric cancer, locally advanced, neoadjuvant chemotherapy, survival, outcomes

## Abstract

**Background:**

Gastric cancer is the second main cause of cancer-related deaths. Since the disease does not produce typical symptoms in the early periods, diagnosis is usually made in locally advanced stages. Although surgery is the most curative treatment, neoadjuvant chemotherapy (NACT) occupies a highly important place in prognosis.

**Methods:**

Patients operated due to locally advanced gastric cancer (LAGC) in 2015-2022 were investigated retrospectively. These were divided into 2 groups, NACT + surgery + adjuvant chemotherapy (AC) (group 1) and surgery-ACT (group 2). The patients’ clinicopathological characteristics and postoperative outcomes were compared. Overall survival (OS) and disease-free survival (DFS) analyses were also performed.

**Results:**

Eighty-four patients who underwent surgery following NACT and 60 resectable patients who underwent surgery and then received ACT were evaluated. The patients’ mean age was 62.96 years, and 61.8% were men. Perineural and lymphovascular invasion, total lymph nodes removed, and numbers of metastatic lymph nodes were significantly higher in group 2 (*P *< .05). Postoperative complication rates were 22.6% in group I and 33.33% in group 2, the difference being statistically significant (*P* = .034). Recurrence and metastasis rates were higher in group 2. Patients in group 1 exhibited significantly longer OS and DFS than those in group 2 (*P *< .05).

**Conclusion:**

Neoadjuvant chemotherapy has a positive impact on survival in LAGC patients. It also reduces recurrence and metastasis rates and postoperative complications. The most important factor affecting survival is the patient’s receipt of NACT. Other factors affecting OS and DFS include metastatic lymph node numbers and lymphovascular invasion.

**Main Points**

- Gastric cancer is the second main cause of cancer-related deaths.
- Neoadjuvant chemotherapy (NACT) emerged as the standard treatment for stage II/III gastric cancers.
- Neoadjuvant chemotherapy has a positive effect on survival and oncological outcomes in patients with LAGC.

## Introduction

Gastric cancer is the second most important cause of cancer-related mortality and the fourth most prevalent form of cancer worldwide.^1,2^ The most important factor affecting prognosis and survival is the disease stage. Due to the few typical symptoms observed in early stage gastric cancer, more than 50% of patients are diagnosed in the locally advanced disease stage.^3^ Locally advanced gastric cancer (LAGC) encapsulates stages T2 and above, irrespective of lymph node involvement.^4^ The most curative treatment in patients with resectable LAGC is surgical resection. However, despite surgical resection (D2 gastrectomy) with lymphadenectomy, local recurrence and distant metastasis rates are still high. The prognosis of LAGC is poor, with 5-year survival rates below 50%.^5,6^ Different therapeutic modalities are therefore needed to increase pre- and postoperative survival.^6^ Although there is no full consensus regarding any therapeutic modality, neoadjuvant therapy (NAT) and adjuvant therapy (AT) together with curative resection constitute a strategy that improves survival.^6^ Postoperative AT is another therapeutic method that enhances survival.^7^

Neoadjuvant chemotherapy (NACT) emerged as the standard treatment for stage II/III gastric cancers following the MAGIC (Medical Research Council Adjuvant Gastric Infusional Chemotherapy) trial study published in 2006.^8^ Although preoperative chemotherapy with R0 resection is the most popular treatment in Europe, postoperative chemoradiation is more widely performed in America, and postoperative chemotherapy in Asia. Chemotherapy, either pre- or postoperative, entails greater survival benefits than surgery alone. Radiotherapy has not yet become a routine treatment for patients with LAGC, and research is still continuing.^6-9^ Although NAT is the most widely employed medical treatment in LAGCs, it is still not routinely employed, with surgery alone being performed in several regimens.^10^ Studies on this subject are urgently required in order to standardize the treatment of gastric cancers.

This retrospective study compared the outcomes of patients receiving NACT-surgery with those of patients receiving surgery-ACT. The aim of this paper is to discuss the effect on resectability of NACT in patients with LAGC, and its effect on postoperative outcomes, particularly survival, in the light of the current literature.

## Material and Methods

Cases of LAGC undergoing curative gastrectomy followed by adjuvant chemotherapy (surgery-ACT) or curative gastrectomy following NACT and adjuvant chemotherapy (NACT-surgery-ACT) at the Atatürk University Research hospital general surgery clinic between 2015 and 2022 were reviewed retrospectively. The patients were assigned to 2 groups, 1 and 2, according to receipt of NACT. The study data were retrieved from the patients’ files and the hospital’s electronic software system.

Patients aged over 18 diagnosed with clinical stage II and III gastric carcinoma (cT2-4a and/or N+) were included in the study. Patients treated for any other malignant disease, with significant accompanying diseases capable of affecting morbidity and mortality, or whose data were unavailable were excluded.

Data for demographic and clinicopathological characteristics, including gender, age, tumor location, degree of tumor differentiation, pathological T stage, and pathological N status, were collected. Postoperative outcomes such as numbers of lymph nodes resected, postoperative complications, recurrence rates, distant metastasis, overall survival (OS), and disease-free survival (DFS) were compared between the groups. Clinical examination, blood tests, upper gastrointestinal endoscopy, endoscopic ultrasound, chemotherapy (CT), positron emission tomography (PET)/CT, magnetic resonance imaging, and laparoscopic staging methods were employed for postoperative patient evaluation.

The cases were staged in line with the TNM staging system for gastric cancer as recommended by the 8th American Joint Committee on Cancer. Tumors were also classified as well-, moderately, or poorly differentiated according to the predominant cell type at histological examination.^11^

Although different treatment regimens are employed for NAT, a FLOT regimen (50 mg/m^2^ docetaxel, 85 mg/m^2^ oxaliplatin, 200 mg/m^2^ leucovorin, and 2600 mg/m^2^ fluorouracil 24-hour infusion) is generally applied for 3-4 cycles.^12,13^ Following NACT, PET-CT radiological images were examined to exclude metastatic disease, and surgical treatment was performed 4-6 weeks after the end of chemotherapy. Postoperative chemotherapy was recommended to all operated patients, and they were referred to the medical oncology unit. Radiotherapy is not used in our routine clinical practice. However, it has recently begun being employed together with chemotherapy in esophagogastric junction adenocarcinomas. In line with the purpose of this study, a small number of patients who underwent radiotherapy were excluded from the research. Approval for this retrospective study was granted by the Atatürk University Faculty of Medicine Institutional Research Ethics Board, Türkiye (no. 02.06.2022, B.30.2.ATA.0.01.00/471). All the participants were enrolled after providing signed informed consent form.

All operations were performed by 3 experienced gastrointestinal surgeons. Subtotal or total gastrectomy was performed together with D2 lymph node dissection, depending on the site of the tumor. Billroth II and Roux-en-Y procedures were performed in case of distal gastrectomy, while Roux-en-Y esophagus-jejunal anastomosis was employed in total gastrectomy. No prophylactic splenectomy was involved in either procedure.

Postoperative complications were scored and classified based on the Clavien–Dindo Classification. Morbidity was defined as all postoperative complications until discharge or up to 30 days.^14^ The patients were followed up postoperatively with periodic physical examinations, blood tests (particularly serum tumor markers), CT scans, and gastrointestinal endoscopy. Three- and 5-year OS and DFS analyses were performed.

### Statistical Analysis

During the statistical analysis, numerical data were expressed as mean and standard deviation and categorical data as numbers and percentages. The distribution of numerical data was analyzed by means of a normality test and histogram graphics.

Numerical data were analyzed in the 2 groups using Student’s *t*-test and the Mann–Whitney *U*-test. Categorical data were analyzed using the chi-square test. Relationships between 2 numerical data were evaluated using Pearson’s correlation analysis. The Kaplan–Meier and log-rank tests were employed for survival analysis. Cox-regression tests were applied in the evaluation of factors affecting survival. OS was calculated from the date of surgery to the date of death or most recent contact, and DFS as the time from surgery to the date of recurrence or metastasis.

SPSS version 23.0 for Windows software (IBM SPSS Corp.; Armonk, NY, USA) was used for data recording and statistical evaluations. *P* values < .05 were regarded as statistically significant.

## Results

Two hundred patients meeting the inclusion criteria and evaluated preoperatively as resectable were operated due to LAGC during the 8-year study period. One hundred consecutive patients received NAT, and the other 100 consecutive patients underwent D2 gastrectomy without NACT. Eighty-four patients (84%) were resectable in group 1 (NACT-surgery-AT) and 60 (60%) in group 2 (surgery-AT). The resectability rate was significantly higher in the patients receiving NACT (*P *< .001). Sixteen patients meeting the inclusion criteria but identified as unresectable in group 1 and 40 in group 2 were excluded from the study. The resectable gastric cancer patients’ demographic and clinicopathological features are summarized in Table 1.

Mean age was 61.12 ± 9.42 in group 1 and 65.55 ± 10.82 in group 2 (*P *= .006). Men represented 59.52% of the patients in group 1 and 65% of those in group 2. Although the number of men were significantly higher in both groups, there was no significant difference between the 2 (*P* = .505) (Table 1).

Three subgroups were established based on the tumor site—proximal (cardia and fundus), middle (corpus), and distal (antrum, pylorus). The cancer was most commonly located in the proximal part (group 1: 61.9%, group 2: 46.67%). According to the degree of differentiation in group 1, 29.76% of tumors were well differentiated, 52.38% were moderately differentiated, and 17.86% were poorly differentiated. In group 2, 1.6% of tumors were well differentiated, 61.67% were moderately differentiated, and 36.7% were poorly differentiated. More poorly differentiated tumors were observed in the surgery-ACT group than in the NACT-surgery group (36.7% vs. 17.85, respectively *P *< .01) (Table 1). The patients in the NACT-surgery group exhibited better tumor differentiation, regarded as linked to a better response to chemotherapy.^15^

Total gastrectomy was performed on 78.57% of the patients in group 1 and on 71.67% of those in group 2. Total gastrectomy was thus performed on the great majority of patients, although there was no significant difference between the 2 groups (*P *= .341) (Table 2).

Histopathological evaluation of materials removed during surgery revealed adenocarcinoma in more than 80% of patients from both groups (82.14% and 87.5%, respectively). This was followed, in decreasing order, by signet ring cell adenocarcinoma, mucinous adenocarcinoma, and mixed (mucinous + signet ring cell) cancers (Table 1).

Perineural and lymphovascular invasion were significantly greater in group 2 (surgery-ACT) (*P* < .001). Total numbers of lymph nodes removed and numbers of metastatic lymph nodes were also higher in the surgery-ACT group (*P* < .05). Patients in group 1 (NACT-surgery) had a lower stage, based on pT and pN stage status (*P* < .001) (Table 2).

Postoperative outcomes are shown in Table 2. The overall morbidity rate was 27%, and complication rates were 19/84 (22.6%) in group 1 and 20/60 (33.33%) in group 2, the difference being statistically significant (*P *= .034). Lengths of hospitalization were 11.74 ± 2.13 days in group 1 and 12.62 ± 2.63 in group 2 (*P *= .029).

Although the recurrence rate was lower in the group receiving NAT, the difference between the groups was not statistically significant (7.14% and 15%, respectively, *P* = .128). The metastasis rate was higher in the surgery-ACT group (33.3% and 11.9%, respectively, *P* = .002) (Table 2). Neoadjuvant therapy particularly lowers metastasis status.

**Figure 1.**
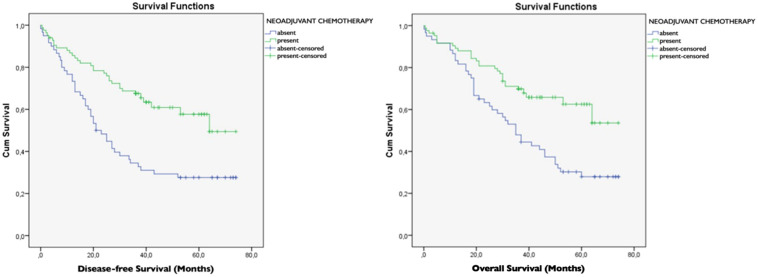
Kaplan-Meier Curves for overall and disease-free survival.

Overall survival was 54.07 ± 3.03 months in group 1 and 39.56 ± 3.3 in group 2 (*P* = .01). Disease-free survival was 51.24 ± 3.18 months in group 1 and 33.33 ± 3.52 in group 2 (*P*< .001) (Figure 1). Patients in group 1 exhibited significantly longer OS and DFS than those in group 2 (*P *< .05). Three-year OS rates were 69.05% in group 1 and 46.67% in group 2 (surgery-ACT) (*P* < .001). The OS rate at a 5-year follow-up was 64.29% in group 1 and 30% in group 2 (surgery-ACT) (*P* < .001). Disease-free survival rates at the 3-year follow-up were 67.86 (n = 57) in group 1 and 35% (n = 21) in group 2 (*P* < .001). Kaplan–Meier survival curves for OS and DFS are shown in Figure 1.

Cox-regression analysis was performed in order to identify factors affecting OS and DFS between the NACT and non-NACT groups. Neoadjuvant chemotherapy emerged as the most important factor affecting OS and DFS. Number of metastatic lymph nodes and lymphovascular invasion also affected OS (*P *< .05). The total number of lymph nodes removed and lymphovascular invasion were factors affecting DFS (*P *< .05) (Table 3).

## Discussion

The prognosis of LAGC treated with surgical resection alone is poor, and the use of NAT has been recommended to enhance oncological and surgical outcomes.^16^ Although different therapeutic regimens are employed in some regions, a combination of D2 gastrectomy and perioperative chemotherapy (PCT) has become the standard treatment. Neoadjuvant therapy is the more important step in this combination.^17^ Neoadjuvant therapy is recommended for potentially resectable patients at T2N0 or more advanced stages.^18^ Neoadjuvant therapy has a number of advantages, such as increasing curative resection rates, preventing micrometastases, reducing the stage in locally advanced tumors, being better tolerated by patients when applied before surgery, and planning AT regimens on the basis of the pathological response.^19^ Charruf et al reported lower pT and pN status and less venous, lymphatic, and perineural invasion in patients receiving NACT. The fact that patients in the PN0 stage who received NAT and those who did not receive it exhibited similar results highlights the importance of stage down.^20^

Consistent with the previous literature, the patient who received NACT in this study had lower pT and pN status and less lymphovascular and perineural invasion. Particularly noteworthy are the pT0 rate of 25% and the pN0 rate (64.29%) in the patients in group 1. These data show the clinical benefit of NACT.

A previous randomized, controlled study showed that PCT increased DFS and OS in patients with LAGC and also raised the curative resection rate.^21^ Resectability in patients receiving NACT exceeds 90%.^17^ Similarly in the present study, NACT not only increased survival but also significantly raised the resectability rate (84%).

Despite all these advantages, there is still a lack of evidence showing that NAT increases postoperative survival compared to AT. Some authors hold the view that NAC increases postoperative complications and has numerous side effects. In contrast to North America and Europe, NAC is not popular in Asia. The effect of NAT is therefore still controversial, and further studies are needed on the subject.^22^ The use of radiotherapy, another component of NAT, is controversial in patients with LAGC. No superiority to chemotherapy has been shown for radiotherapy in terms of OS or DFS, although research into radiotherapy is continuing.^23^ Although radiotherapy has been employed in a few patients in our own clinical practice, it has not become a routine treatment. While AT (surgery-AT) was administered for patients with LAGC. in the early years after 2015, perioperative chemotherapy (NACT-surgery-AT) has now become a routine treatment.

Although NACT increases the rate of complications due to the adverse effects of chemotherapy in the postoperative period, we think that it reduces postoperative complication rates due to improved nutritional status and a decreased tumor burden after NACT. Inconsistent results have been reported in the literature.^17-20^ One recent study reported total postoperative complication rates of 24.8% and 19.8% for NACT-S and surgery-ACT groups (*P *= .207), respectively.^22^ Another study reported a lower postoperative complication rate in a group receiving NACT (6.7% vs. 24.4%), and a shorter length of hospitalization in that group.^20^

Postoperative complication rates in the present study were lower in the NACT group (*P *< .034) (22.6% compared to 33.33%), and the NACT group also exhibited a shorter length of hospitalization (*P* = .029).

Since NACT lowers the tumor stage, patients require less aggressive surgery, thus resulting in fewer complications. Neoadjuvant chemotherapy particularly reduces major complications, and naturally shortens lengths of stay. Neoadjuvant chemotherapy naturally positively affected OS and DFS by reducing tumor size, lymphatic, neural, and venous invasion, resulting in fewer metastatic lymph nodes.^17,20,24^ Studies have reported 5-year OS rates of 60.9-72.29% (NAT group) and 45.9-49.8% (surgery-AT).^17,22^

In the present study, 5-year OS rates during 5-year follow-up were 64.29% in group 1 and 30% in group 2 (AT plus surgery). Consistent with the previous literature, NACT improved OS better than AT.

In addition, patients in the neoadjuvant chemotherapy plus surgery plus adjuvant chemotherapy group achieved an improvement in 3-year DFS (64.6% vs. 58.0%).^22^ The PRODIGY study revealed significantly higher 3- and 5-year DFS rates in the NAC + surgery + adjuvant chemotherapy group than those in the surgery + adjuvant chemotherapy group.^25^ Three-year DFS rates were 69.74% in the NAT plus surgery group and 39.86% in the surgery group.^17^ Disease-free survival rates over a 3-year follow-up in the present study were 67.86% (n = 57) and 35% (n =21), respectively. Although NAT has been shown to increase DFS by approximately 8% compared to adjuvant chemotherapy in the previous literature, the equivalent figure in the present research was approximately 33%. We, therefore, concluded that NAT significantly improves DFS.

The number of studies using multivariate analysis to examine the factors affecting survival is relatively small, and the data yielded on the subject are important.^24^ Although tumor regression does affect prognosis, N status has been described as a more important prognostic factor. In that recent study, ypT4 status emerged as an independent predictor of recurrence.^26^ Diffuse type histology that determines tumor behavior is one of the most important factors adversely affecting survival.^24^ In the present study, NACT emerged as one of the most important factors affecting both OS and DFS. Metastatic lymph node numbers and lymphovascular invasion are also factors that affect OS (*P* < .05). The number of lymph nodes extracted, the number of metastatic lymph nodes, and lymphovascular invasion affect DFS.

### Limitations of the Study

There are a number of strengths and limitations to this study. The principal limitation involves its retrospective nature. The compatibility between preoperative clinical TNM staging and postoperative pathological staging, and also re- and post-chemotherapy clinical losses could not be compared. In addition, the patient group receiving surgery alone could not be compared with patient groups receiving chemotherapy. Further prospective, randomized, controlled clinical trials are now needed on this subject. Nonetheless, despite these limitations, this study reports data from a significant number of patients with gastric cancer, a disease with high morbidity and mortality rates. The application of multivariate analysis represents another strength of this research.

In conclusion, NACT has a positive effect on survival in patients with LAGC. It reduces recurrence and metastasis rates and postoperative complications. NACT represents a safe, effective, and practical modality in terms of both surgical and oncological outcomes. The multivariate analysis results identified receipt of NACT as the most important factor affecting survival. The number of metastatic lymph nodes and lymphovascular invasion affect both OS and DFS.

## Figures and Tables

**Table 1.** Patient Demographic Features and Clinicopathological Characteristics

*Variables*	Group 1 (NACT + Surgery +/− AC)	Group 2 (Surgery-ACT)	*P*
	n = 84 (%)	n = 60 (%)	
Age*	61.12 ± 9.42	65.55 ± 10.82	.006
Gender*			
Female/Male	34/50 (40.48)	21/39 (35)	.505
Tumor location			
Proximal	52 (61.9)	28 (46.67)	
Middle	10 (11.9)	15 (25)	
Distal	22 (26.2)	17 (28.33)	
Degree of differentiation*			
Well differentiation	25 (29.76)	1 (1.67)	
Moderately differentiation	44 (52.38)	37 (61.67)	
Poorly differentiation	15 (17.85)	22 (36.7)	<.001
Histopathology*			
Adenocarcinoma	69 (82.14)	35 (58.33)	
Signet ring cell adenocarcinoma	7 (8.33)	19 (31.66)	
Mucinous adenocarcinoma	5 (5.95)	5 (8.33)	
Mixed (mucinous + signet ring cell)	3 (3.57)	1 (1.66)	0.003
Length of hospital stay*	11.74 ± 2.13	12.62 ± 2.63	0.029

AC, adjuvant chemotherapy; NACT, neoadjuvant chemotherapy.

**Table 2.** Operative Data and Postoperative Results of Patients

*Variables*	Group 1 (NACT + Surgery +/− AC)	Group 2 (Surgery-ACT)	*P*
	n = 84 (%)	n = 60 (%)	
Extent of resection*			
Distal subtotal	18 (21.42)	17 (28.33)	
Total	66 (78.57)	43 (71.67)	.341
Pathological T stage*			
T0	21 (25)	0 (0)	
T1	8 (9.52)	2 (3.33)	
T2	8 (9.52)	5 (8.33)	
T3	26 (30.95)	11 (18.33)	
T4	21 (25)	42 (70)	<.001
Pathological N stage*			
cN0	54 (64.29)	9 (15)	
cN1	17 (20.24)	16 (26.67)	
cN2	6 (7.14)	7 (11.67)	
cN3	7 (8.33)	28 (46.67)	<.001
Lymphovascular invasion*			
Absent	44 (52.38)	5 (8.33)	
Present	40 (47.62)	55 (91.67)	<.001
Perineural invasion*			
Absent	48 (57.14)	5 (8.33)	
Present	36 (42.86)	55 (91.67)	<.001
Metastasis*			
Absent	74 (88.10)	40 (66.67)	
Present	10 (11.90)	20 (33.33)	.002
Recurrence*			
Absent	78 (92.86)	51 (85)	
Present	6 (7.14)	9 (15)	.128
Postoperative complications			
CD1	0 (0)	1 (1.66)	
CD2	5 (5.95)	12 (20)	
CD3	12 (14.2)	6 (10)	
CD4	2 (2.38)	1 (1.66)	.034

AC, adjuvant chemotherapy; CD, Clavien–Dindo; NACT, neoadjuvant chemotherapy.

**Table 3.** Parameters Affecting Overall and Disease-free Survival, Multivariate Logistic Regression Analysis

	Overall Survival					Disease‐free Survival				
	B	SE	Wald	*P*	Exp(B)	B	SE	Wald	*P*	Exp(B)
Tumor location			0.465	.792				0.263	.877	
Proximal	0.177	0.294	0.364	.547	0.837	0.048	0.284	0.029	.865	0.953
Middle-distal	0.214	0.373	0.331	.565	0.807	0.188	0.374	0.254	.614	0.828
Histopathology			2.948	.400				3.466	.325	
Adenocarcinoma	0.029	0.755	0.001	.969	0.971	0.545	0.639	0.726	.394	0.580
Signet ring cell adenocarcinoma	0.547	0.824	0.440	.507	0.579	0.928	0.710	1.710	.191	0.395
Mucinous adenocarcinoma	0.335	0.864	0.150	.699	1.397	0.073	0.749	0.009	.922	0.930
Differentiation			3.109	.211				1.752	.416	
Poor	0.353	0.568	0.386	.534	0.703	0.011	0.526	0.000	.983	1.011
Well-moderate	0.196	0.540	0.132	.717	1.216	0.379	0.506	0.561	.454	1.461
Total lymph nodes	0.017	0.013	1.687	.194	1.017	0.032	0.015	4.583	.032	1.033
Metastatic lymph nodes	0.033	0.014	5.317	.021	0.968	0.051	0.016	10.200	.001	0.950
Length of hospital stay	0.001	0.059	0.001	.981	1.001	0.020	0.057	0.116	.733	0.981
Lymphovascular invasion	1.371	0.559	6.020	.014	0.254	1.301	0.536	5.891	.015	0.272
Perineural invasion	0.047	0.509	0.009	.926	1.048	0.151	0.482	0.098	.754	1.163
